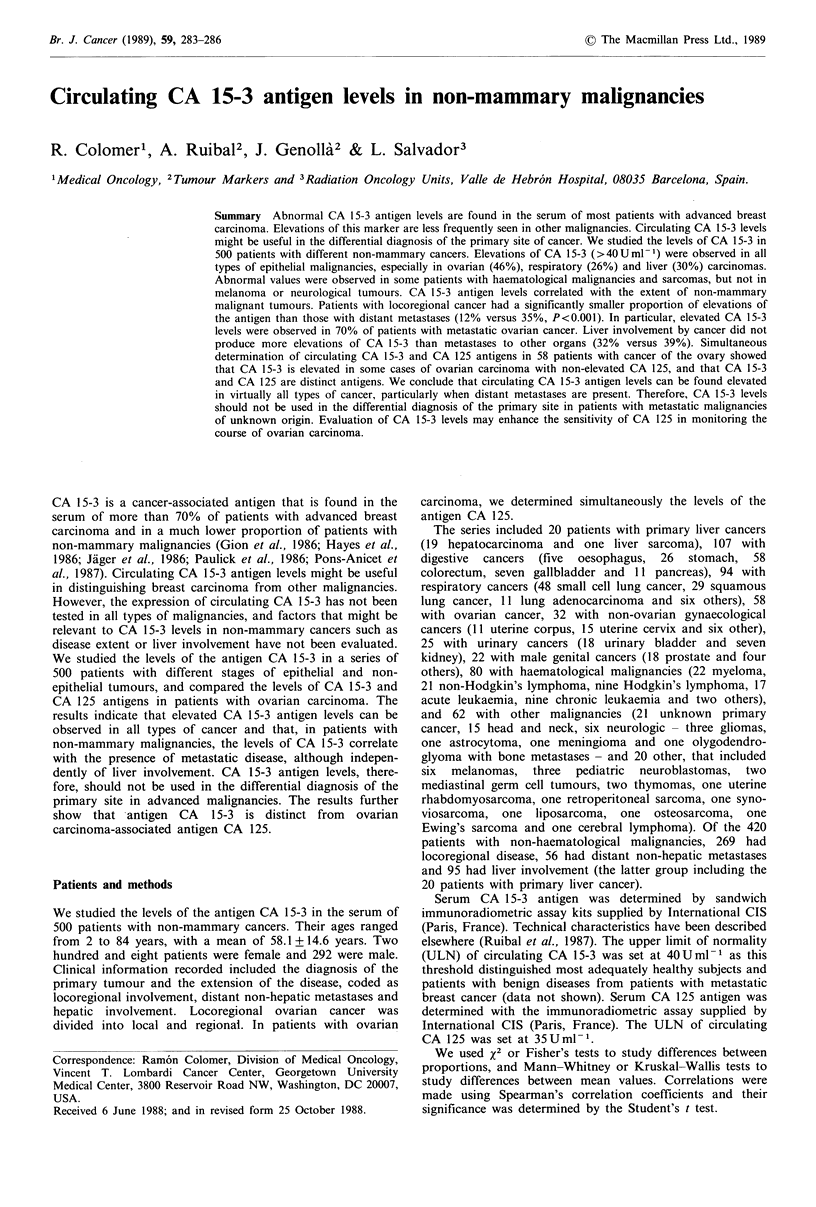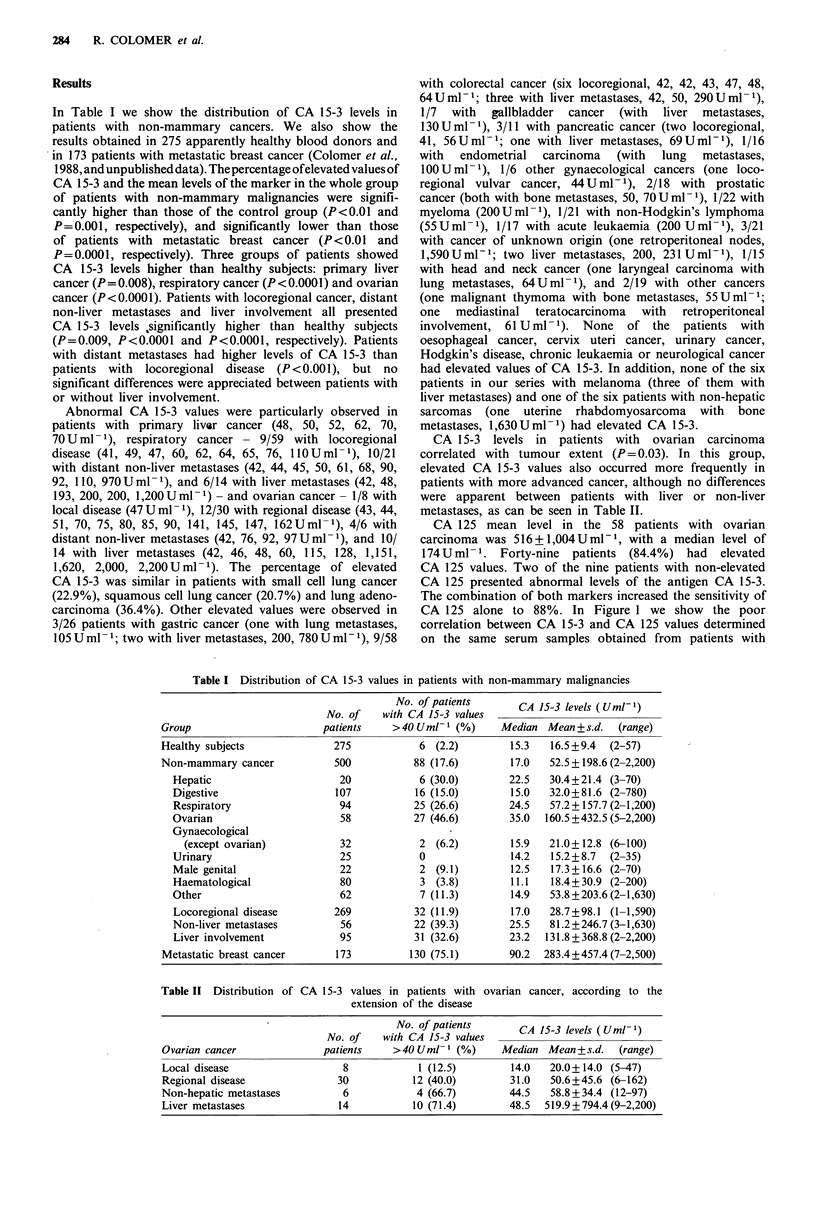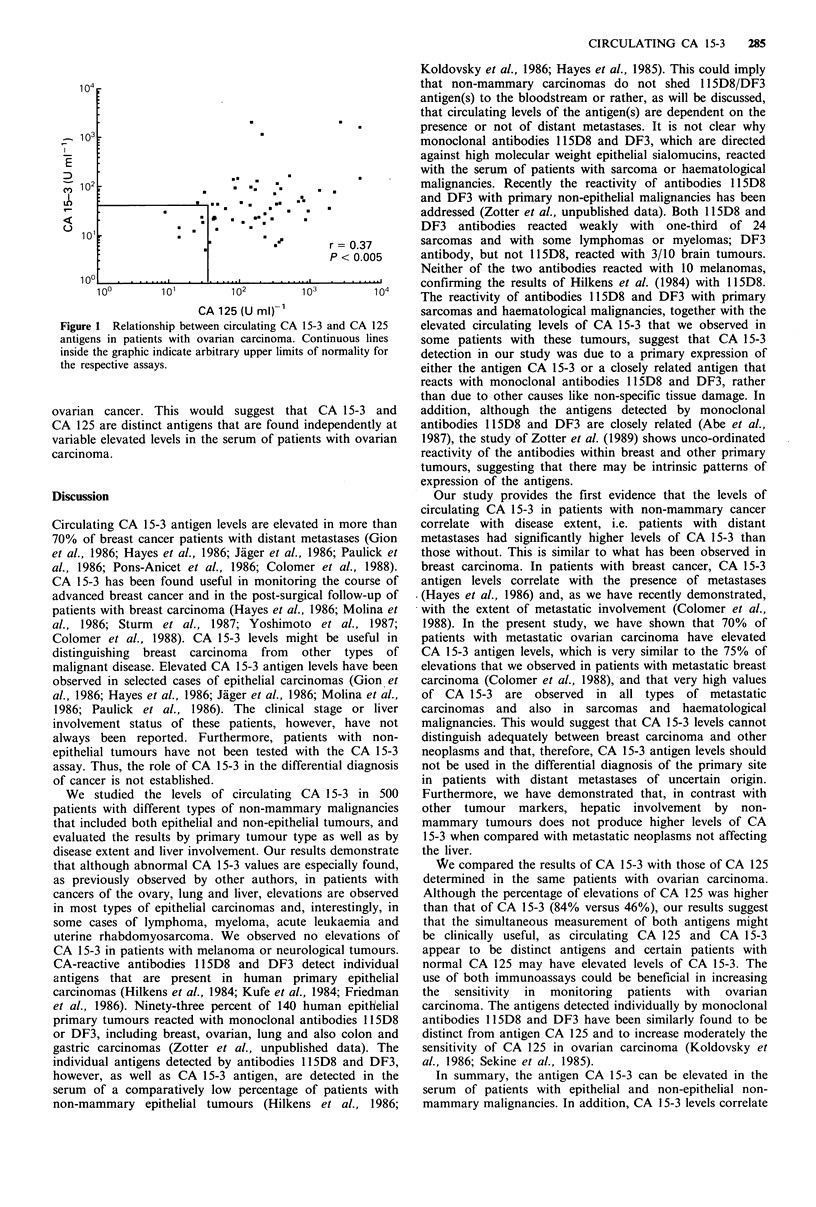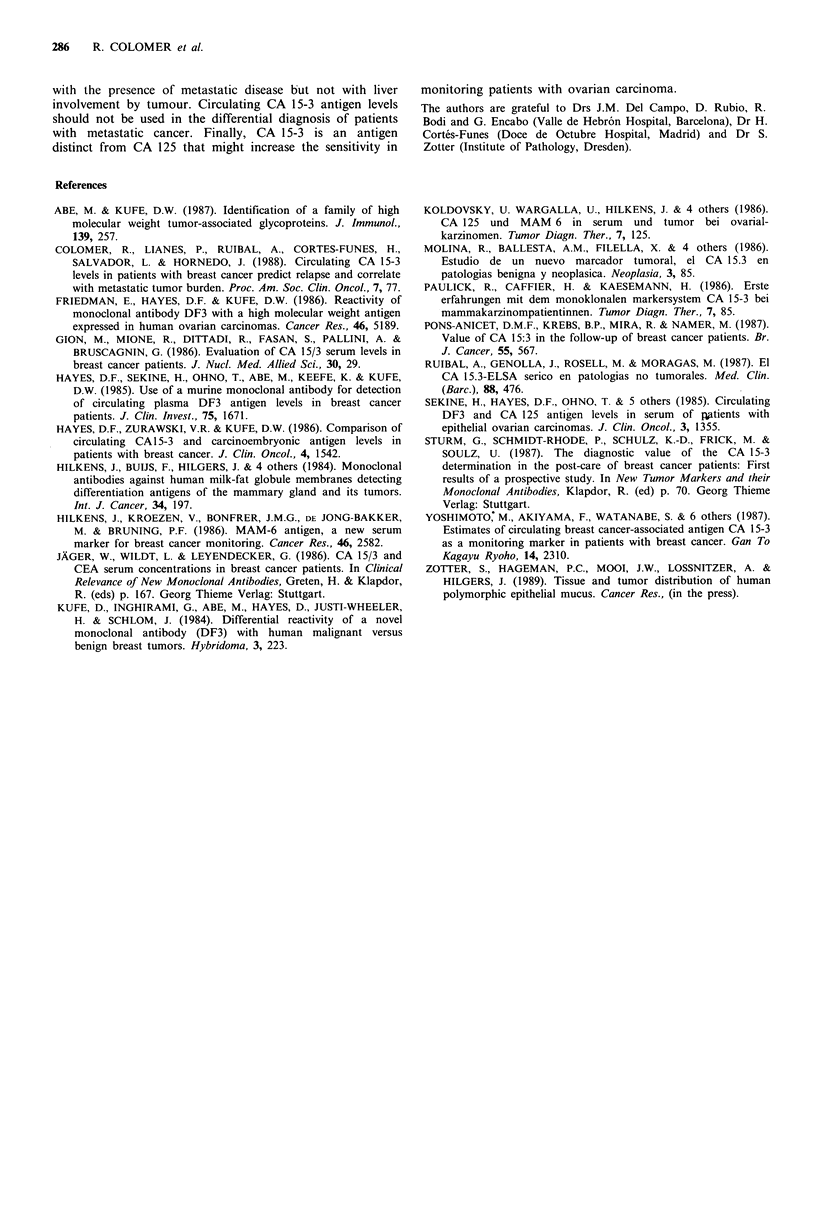# Circulating CA 15-3 antigen levels in non-mammary malignancies.

**DOI:** 10.1038/bjc.1989.58

**Published:** 1989-02

**Authors:** R. Colomer, A. Ruibal, J. GenollÃ , L. Salvador

**Affiliations:** Medical Oncology Unit, Valle de HebrÃ³n Hospital, Barcelona, Spain.

## Abstract

Abnormal CA 15-3 antigen levels are found in the serum of most patients with advanced breast carcinoma. Elevations of this marker are less frequently seen in other malignancies. Circulating CA 15-3 levels might be useful in the differential diagnosis of the primary site of cancer. We studied the levels of CA 15-3 in 500 patients with different non-mammary cancers. Elevations of CA 15-3 (greater than 40 U ml-1) were observed in all types of epithelial malignancies, especially in ovarian (46%), respiratory (26%) and liver (30%) carcinomas. Abnormal values were observed in some patients with haematological malignancies and sarcomas, but not in melanoma or neurological tumours. CA 15-3 antigen levels correlated with the extent of non-mammary malignant tumours. Patients with locoregional cancer had a significantly smaller proportion of elevations of the antigen than those with distant metastases (12% versus 35%, P less than 0.001). In particular, elevated CA 15-3 levels were observed in 70% of patients with metastatic ovarian cancer. Liver involvement by cancer did not produce more elevations of CA 15-3 than metastases to other organs (32% versus 39%). Simultaneous determination of circulating CA 15-3 and CA 125 antigens in 58 patients with cancer of the ovary showed that CA 15-3 is elevated in some cases of ovarian carcinoma with non-elevated CA 125, and that CA 15-3 and CA 125 are distinct antigens. We conclude that circulating CA 15-3 antigen levels can be found elevated in virtually all types of cancer, particularly when distant metastases are present. Therefore, CA 15-3 levels should not be used in the differential diagnosis of the primary site in patients with metastatic malignancies of unknown origin. Evaluation of CA 15-3 levels may enhance the sensitivity of CA 125 in monitoring the course of ovarian carcinoma.


*Br. J. Cancer* (1989), **59**, 283–286 © The Macmillan Press Ltd., 1989

### Circulating CA 15-3 antigen levels in non-mammary malignancies

R. Colomer[1], A. Ruibal[2], J. Genollà[2] & L. Salvador[3]

[1]*Medical Oncology,* [2]*Tumour Markers and* [3]*Radiation Oncology Units, Valle de Hebrón Hospital, 08035 Barcelona, Spain.*

**Summary** Abnormal CA 15-3 antigen levels are found in the serum of most patients with advanced breast carcinoma. Elevations of this marker are less frequently seen in other malignancies. Circulating CA 15-3 levels might be useful in the differential diagnosis of the primary site of cancer. We studied the levels of CA 15-3 in 500 patients with different non-mammary cancers. Elevations of CA 15-3 ($>40\,U\,ml^{-1}$) were observed in all types of epithelial malignancies, especially in ovarian (46%), respiratory (26%) and liver (30%) carcinomas. Abnormal values were observed in some patients with haematological malignancies and sarcomas, but not in melanoma or neurological tumours. CA 15-3 antigen levels correlated with the extent of non-mammary malignant tumours. Patients with locoregional cancer had a significantly smaller proportion of elevations of the antigen than those with distant metastases (12% versus 35%, $P<0.001$). In particular, elevated CA 15-3 levels were observed in 70% of patients with metastatic ovarian cancer. Liver involvement by cancer did not produce more elevations of CA 15-3 than metastases to other organs (32% versus 39%). Simultaneous determination of circulating CA 15-3 and CA 125 antigens in 58 patients with cancer of the ovary showed that CA 15-3 is elevated in some cases of ovarian carcinoma with non-elevated CA 125, and that CA 15-3 and CA 125 are distinct antigens. We conclude that circulating CA 15-3 antigen levels can be found elevated in virtually all types of cancer, particularly when distant metastases are present. Therefore, CA 15-3 levels should not be used in the differential diagnosis of the primary site in patients with metastatic malignancies of unknown origin. Evaluation of CA 15-3 levels may enhance the sensitivity of CA 125 in monitoring the course of ovarian carcinoma.

CA 15-3 is a cancer-associated antigen that is found in the serum of more than 70% of patients with advanced breast carcinoma and in a much lower proportion of patients with non-mammary malignancies (Gion *et al.*, 1986; Hayes *et al.*, 1986; Jäger *et al.*, 1986; Paulick *et al.*, 1986; Pons-Anicet *et al.*, 1987). Circulating CA 15-3 antigen levels might be useful in distinguishing breast carcinoma from other malignancies. However, the expression of circulating CA 15-3 has not been tested in all types of malignancies, and factors that might be relevant to CA 15-3 levels in non-mammary cancers such as disease extent or liver involvement have not been evaluated. We studied the levels of the antigen CA 15-3 in a series of 500 patients with different stages of epithelial and non-epithelial tumours, and compared the levels of CA 15-3 and CA 125 antigens in patients with ovarian carcinoma. The results indicate that elevated CA 15-3 antigen levels can be observed in all types of cancer and that, in patients with non-mammary malignancies, the levels of CA 15-3 correlate with the presence of metastatic disease, although independently of liver involvement. CA 15-3 antigen levels, therefore, should not be used in the differential diagnosis of the primary site in advanced malignancies. The results further show that antigen CA 15-3 is distinct from ovarian carcinoma-associated antigen CA 125.

### Patients and methods

We studied the levels of the antigen CA 15-3 in the serum of 500 patients with non-mammary cancers. Their ages ranged from 2 to 84 years, with a mean of $58.1 \pm 14.6$ years. Two hundred and eight patients were female and 292 were male. Clinical information recorded included the diagnosis of the primary tumour and the extension of the disease, coded as locoregional involvement, distant non-hepatic metastases and hepatic involvement. Locoregional ovarian cancer was divided into local and regional. In patients with ovarian carcinoma, we determined simultaneously the levels of the antigen CA 125.

The series included 20 patients with primary liver cancers (19 hepatocarcinoma and one liver sarcoma), 107 with digestive cancers (five oesophagus, 26 stomach, 58 colorectum, seven gallbladder and 11 pancreas), 94 with respiratory cancers (48 small cell lung cancer, 29 squamous lung cancer, 11 lung adenocarcinoma and six others), 58 with ovarian cancer, 32 with non-ovarian gynaecological cancers (11 uterine corpus, 15 uterine cervix and six other), 25 with urinary cancers (18 urinary bladder and seven kidney), 22 with male genital cancers (18 prostate and four others), 80 with haematological malignancies (22 myeloma, 21 non-Hodgkin's lymphoma, nine Hodgkin's lymphoma, 17 acute leukaemia, nine chronic leukaemia and two others), and 62 with other malignancies (21 unknown primary cancer, 15 head and neck, six neurologic – three gliomas, one astrocytoma, one meningioma and one olygodendroglyoma with bone metastases – and 20 other, that included six melanomas, three pediatric neuroblastomas, two mediastinal germ cell tumours, two thymomas, one uterine rhabdomyosarcoma, one retroperitoneal sarcoma, one synoviosarcoma, one liposarcoma, one osteosarcoma, one Ewing's sarcoma and one cerebral lymphoma). Of the 420 patients with non-haematological malignancies, 269 had locoregional disease, 56 had distant non-hepatic metastases and 95 had liver involvement (the latter group including the 20 patients with primary liver cancer).

Serum CA 15-3 antigen was determined by sandwich immunoradiometric assay kits supplied by International CIS (Paris, France). Technical characteristics have been described elsewhere (Ruibal *et al.*, 1987). The upper limit of normality (ULN) of circulating CA 15-3 was set at $40\,U\,ml^{-1}$ as this threshold distinguished most adequately healthy subjects and patients with benign diseases from patients with metastatic breast cancer (data not shown). Serum CA 125 antigen was determined with the immunoradiometric assay supplied by International CIS (Paris, France). The ULN of circulating CA 125 was set at $35\,U\,ml^{-1}$.

We used $\chi^2$ or Fisher's tests to study differences between proportions, and Mann–Whitney or Kruskal–Wallis tests to study differences between mean values. Correlations were made using Spearman's correlation coefficients and their significance was determined by the Student's $t$ test.

Correspondence: Ramón Colomer, Division of Medical Oncology, Vincent T. Lombardi Cancer Center, Georgetown University Medical Center, 3800 Reservoir Road NW, Washington, DC 20007, USA.
Received 6 June 1988; and in revised form 25 October 1988.

### Results

In Table I we show the distribution of CA 15-3 levels in patients with non-mammary cancers. We also show the results obtained in 275 apparently healthy blood donors and in 173 patients with metastatic breast cancer (Colomer *et al.*, 1988, and unpublished data). The percentage of elevated values of CA 15-3 and the mean levels of the marker in the whole group of patients with non-mammary malignancies were significantly higher than those of the control group ($P<0.01$ and $P=0.001$, respectively), and significantly lower than those of patients with metastatic breast cancer ($P<0.01$ and $P=0.0001$, respectively). Three groups of patients showed CA 15-3 levels higher than healthy subjects: primary liver cancer ($P=0.008$), respiratory cancer ($P<0.0001$) and ovarian cancer ($P<0.0001$). Patients with locoregional cancer, distant non-liver metastases and liver involvement all presented CA 15-3 levels significantly higher than healthy subjects ($P=0.009$, $P<0.0001$ and $P<0.0001$, respectively). Patients with distant metastases had higher levels of CA 15-3 than patients with locoregional disease ($P<0.001$), but no significant differences were appreciated between patients with or without liver involvement.

Abnormal CA 15-3 values were particularly observed in patients with primary liver cancer (48, 50, 52, 62, 70, 70 U ml$^{-1}$), respiratory cancer – 9/59 with locoregional disease (41, 49, 47, 60, 62, 64, 65, 76, 110 U ml$^{-1}$), 10/21 with distant non-liver metastases (42, 44, 45, 50, 61, 68, 90, 92, 110, 970 U ml$^{-1}$), and 6/14 with liver metastases (42, 48, 193, 200, 200, 1,200 U ml$^{-1}$) – and ovarian cancer – 1/8 with local disease (47 U ml$^{-1}$), 12/30 with regional disease (43, 44, 51, 70, 75, 80, 85, 90, 141, 145, 147, 162 U ml$^{-1}$), 4/6 with distant non-liver metastases (42, 76, 92, 97 U ml$^{-1}$), and 10/14 with liver metastases (42, 46, 48, 60, 115, 128, 1,151, 1,620, 2,000, 2,200 U ml$^{-1}$). The percentage of elevated CA 15-3 was similar in patients with small cell lung cancer (22.9%), squamous cell lung cancer (20.7%) and lung adenocarcinoma (36.4%). Other elevated values were observed in 3/26 patients with gastric cancer (one with lung metastases, 105 U ml$^{-1}$; two with liver metastases, 200, 780 U ml$^{-1}$), 9/58 with colorectal cancer (six locoregional, 42, 42, 43, 47, 48, 64 U ml$^{-1}$; three with liver metastases, 42, 50, 290 U ml$^{-1}$), 1/7 with gallbladder cancer (with liver metastases, 130 U ml$^{-1}$), 3/11 with pancreatic cancer (two locoregional, 41, 56 U ml$^{-1}$; one with liver metastases, 69 U ml$^{-1}$), 1/16 with endometrial carcinoma (with lung metastases, 100 U ml$^{-1}$), 1/6 other gynaecological cancers (one locoregional vulvar cancer, 44 U ml$^{-1}$), 2/18 with prostatic cancer (both with bone metastases, 50, 70 U ml$^{-1}$), 1/22 with myeloma (200 U ml$^{-1}$), 1/21 with non-Hodgkin's lymphoma (55 U ml$^{-1}$), 1/17 with acute leukaemia (200 U ml$^{-1}$), 3/21 with cancer of unknown origin (one retroperitoneal nodes, 1,590 U ml$^{-1}$; two liver metastases, 200, 231 U ml$^{-1}$), 1/15 with head and neck cancer (one laryngeal carcinoma with lung metastases, 64 U ml$^{-1}$), and 2/19 with other cancers (one malignant thymoma with bone metastases, 55 U ml$^{-1}$; one mediastinal teratocarcinoma with retroperitoneal involvement, 61 U ml$^{-1}$). None of the patients with oesophageal cancer, cervix uteri cancer, urinary cancer, Hodgkin's disease, chronic leukaemia or neurological cancer had elevated values of CA 15-3. In addition, none of the six patients in our series with melanoma (three of them with liver metastases) and one of the six patients with non-hepatic sarcomas (one uterine rhabdomyosarcoma with bone metastases, 1,630 U ml$^{-1}$) had elevated CA 15-3.

CA 15-3 levels in patients with ovarian carcinoma correlated with tumour extent ($P=0.03$). In this group, elevated CA 15-3 values also occurred more frequently in patients with more advanced cancer, although no differences were apparent between patients with liver or non-liver metastases, as can be seen in Table II.

CA 125 mean level in the 58 patients with ovarian carcinoma was $516\pm1,004$ U ml$^{-1}$, with a median level of 174 U ml$^{-1}$. Forty-nine patients (84.4%) had elevated CA 125 values. Two of the nine patients with non-elevated CA 125 presented abnormal levels of the antigen CA 15-3. The combination of both markers increased the sensitivity of CA 125 alone to 88%. In Figure 1 we show the poor correlation between CA 15-3 and CA 125 values determined on the same serum samples obtained from patients with

**Table I** Distribution of CA 15-3 values in patients with non-mammary malignancies

| Group | No. of patients | No. of patients with CA 15-3 values >40 U ml$^{-1}$ (%) | CA 15-3 levels (U ml$^{-1}$) Median | Mean±s.d. | (range) |
|---|---|---|---|---|---|
| Healthy subjects | 275 | 6 (2.2) | 15.3 | 16.5±9.4 | (2–57) |
| Non-mammary cancer | 500 | 88 (17.6) | 17.0 | 52.5±198.6 | (2–2,200) |
| Hepatic | 20 | 6 (30.0) | 22.5 | 30.4±21.4 | (3–70) |
| Digestive | 107 | 16 (15.0) | 15.0 | 32.0±81.6 | (2–780) |
| Respiratory | 94 | 25 (26.6) | 24.5 | 57.2±157.7 | (2–1,200) |
| Ovarian | 58 | 27 (46.6) | 35.0 | 160.5±432.5 | (5–2,200) |
| Gynaecological (except ovarian) | 32 | 2 (6.2) | 15.9 | 21.0±12.8 | (6–100) |
| Urinary | 25 | 0 | 14.2 | 15.2±8.7 | (2–35) |
| Male genital | 22 | 2 (9.1) | 12.5 | 17.3±16.6 | (2–70) |
| Haematological | 80 | 3 (3.8) | 11.1 | 18.4±30.9 | (2–200) |
| Other | 62 | 7 (11.3) | 14.9 | 53.8±203.6 | (2–1,630) |
| Locoregional disease | 269 | 32 (11.9) | 17.0 | 28.7±98.1 | (1–1,590) |
| Non-liver metastases | 56 | 22 (39.3) | 25.5 | 81.2±246.7 | (3–1,630) |
| Liver involvement | 95 | 31 (32.6) | 23.2 | 131.8±368.8 | (2–2,200) |
| Metastatic breast cancer | 173 | 130 (75.1) | 90.2 | 283.4±457.4 | (7–2,500) |

**Table II** Distribution of CA 15-3 values in patients with ovarian cancer, according to the extension of the disease

| Ovarian cancer | No. of patients | No. of patients with CA 15-3 values >40 U ml$^{-1}$ (%) | CA 15-3 levels (U ml$^{-1}$) Median | Mean±s.d. | (range) |
|---|---|---|---|---|---|
| Local disease | 8 | 1 (12.5) | 14.0 | 20.0±14.0 | (5–47) |
| Regional disease | 30 | 12 (40.0) | 31.0 | 50.6±45.6 | (6–162) |
| Non-hepatic metastases | 6 | 4 (66.7) | 44.5 | 58.8±34.4 | (12–97) |
| Liver metastases | 14 | 10 (71.4) | 48.5 | 519.9±794.4 | (9–2,200) |

**Figure 1** Relationship between circulating CA 15-3 and CA 125 antigens in patients with ovarian carcinoma. Continuous lines inside the graphic indicate arbitrary upper limits of normality for the respective assays.

ovarian cancer. This would suggest that CA 15-3 and CA 125 are distinct antigens that are found independently at variable elevated levels in the serum of patients with ovarian carcinoma.

### Discussion

Circulating CA 15-3 antigen levels are elevated in more than 70% of breast cancer patients with distant metastases (Gion *et al.*, 1986; Hayes *et al.*, 1986; Jäger *et al.*, 1986; Paulick *et al.*, 1986; Pons-Anicet *et al.*, 1986; Colomer *et al.*, 1988). CA 15-3 has been found useful in monitoring the course of advanced breast cancer and in the post-surgical follow-up of patients with breast carcinoma (Hayes *et al.*, 1986; Molina *et al.*, 1986; Sturm *et al.*, 1987; Yoshimoto *et al.*, 1987; Colomer *et al.*, 1988). CA 15-3 levels might be useful in distinguishing breast carcinoma from other types of malignant disease. Elevated CA 15-3 antigen levels have been observed in selected cases of epithelial carcinomas (Gion *et al.*, 1986; Hayes *et al.*, 1986; Jäger *et al.*, 1986; Molina *et al.*, 1986; Paulick *et al.*, 1986). The clinical stage or liver involvement status of these patients, however, have not always been reported. Furthermore, patients with non-epithelial tumours have not been tested with the CA 15-3 assay. Thus, the role of CA 15-3 in the differential diagnosis of cancer is not established.

We studied the levels of circulating CA 15-3 in 500 patients with different types of non-mammary malignancies that included both epithelial and non-epithelial tumours, and evaluated the results by primary tumour type as well as by disease extent and liver involvement. Our results demonstrate that although abnormal CA 15-3 values are especially found, as previously observed by other authors, in patients with cancers of the ovary, lung and liver, elevations are observed in most types of epithelial carcinomas and, interestingly, in some cases of lymphoma, myeloma, acute leukaemia and uterine rhabdomyosarcoma. We observed no elevations of CA 15-3 in patients with melanoma or neurological tumours. CA-reactive antibodies 115D8 and DF3 detect individual antigens that are present in human primary epithelial carcinomas (Hilkens *et al.*, 1984; Kufe *et al.*, 1984; Friedman *et al.*, 1986). Ninety-three percent of 140 human epithelial primary tumours reacted with monoclonal antibodies 115D8 or DF3, including breast, ovarian, lung and also colon and gastric carcinomas (Zotter *et al.*, unpublished data). The individual antigens detected by antibodies 115D8 and DF3, however, as well as CA 15-3 antigen, are detected in the serum of a comparatively low percentage of patients with non-mammary epithelial tumours (Hilkens *et al.*, 1986; Koldovsky *et al.*, 1986; Hayes *et al.*, 1985). This could imply that non-mammary carcinomas do not shed 115D8/DF3 antigen(s) to the bloodstream or rather, as will be discussed, that circulating levels of the antigen(s) are dependent on the presence or not of distant metastases. It is not clear why monoclonal antibodies 115D8 and DF3, which are directed against high molecular weight epithelial sialomucins, reacted with the serum of patients with sarcoma or haematological malignancies. Recently the reactivity of antibodies 115D8 and DF3 with primary non-epithelial malignancies has been addressed (Zotter *et al.*, unpublished data). Both 115D8 and DF3 antibodies reacted weakly with one-third of 24 sarcomas and with some lymphomas or myelomas; DF3 antibody, but not 115D8, reacted with 3/10 brain tumours. Neither of the two antibodies reacted with 10 melanomas, confirming the results of Hilkens *et al.* (1984) with 115D8. The reactivity of antibodies 115D8 and DF3 with primary sarcomas and haematological malignancies, together with the elevated circulating levels of CA 15-3 that we observed in some patients with these tumours, suggest that CA 15-3 detection in our study was due to a primary expression of either the antigen CA 15-3 or a closely related antigen that reacts with monoclonal antibodies 115D8 and DF3, rather than due to other causes like non-specific tissue damage. In addition, although the antigens detected by monoclonal antibodies 115D8 and DF3 are closely related (Abe *et al.*, 1987), the study of Zotter *et al.* (1989) shows unco-ordinated reactivity of the antibodies within breast and other primary tumours, suggesting that there may be intrinsic patterns of expression of the antigens.

Our study provides the first evidence that the levels of circulating CA 15-3 in patients with non-mammary cancer correlate with disease extent, i.e. patients with distant metastases had significantly higher levels of CA 15-3 than those without. This is similar to what has been observed in breast carcinoma. In patients with breast cancer, CA 15-3 antigen levels correlate with the presence of metastases (Hayes *et al.*, 1986) and, as we have recently demonstrated, with the extent of metastatic involvement (Colomer *et al.*, 1988). In the present study, we have shown that 70% of patients with metastatic ovarian carcinoma have elevated CA 15-3 antigen levels, which is very similar to the 75% of elevations that we observed in patients with metastatic breast carcinoma (Colomer *et al.*, 1988), and that very high values of CA 15-3 are observed in all types of metastatic carcinomas and also in sarcomas and haematological malignancies. This would suggest that CA 15-3 levels cannot distinguish adequately between breast carcinoma and other neoplasms and that, therefore, CA 15-3 antigen levels should not be used in the differential diagnosis of the primary site in patients with distant metastases of uncertain origin. Furthermore, we have demonstrated that, in contrast with other tumour markers, hepatic involvement by non-mammary tumours does not produce higher levels of CA 15-3 when compared with metastatic neoplasms not affecting the liver.

We compared the results of CA 15-3 with those of CA 125 determined in the same patients with ovarian carcinoma. Although the percentage of elevations of CA 125 was higher than that of CA 15-3 (84% versus 46%), our results suggest that the simultaneous measurement of both antigens might be clinically useful, as circulating CA 125 and CA 15-3 appear to be distinct antigens and certain patients with normal CA 125 may have elevated levels of CA 15-3. The use of both immunoassays could be beneficial in increasing the sensitivity in monitoring patients with ovarian carcinoma. The antigens detected individually by monoclonal antibodies 115D8 and DF3 have been similarly found to be distinct from antigen CA 125 and to increase moderately the sensitivity of CA 125 in ovarian carcinoma (Koldovsky *et al.*, 1986; Sekine *et al.*, 1985).

In summary, the antigen CA 15-3 can be elevated in the serum of patients with epithelial and non-epithelial non-mammary malignancies. In addition, CA 15-3 levels correlate with the presence of metastatic disease but not with liver involvement by tumour. Circulating CA 15-3 antigen levels should not be used in the differential diagnosis of patients with metastatic cancer. Finally, CA 15-3 is an antigen distinct from CA 125 that might increase the sensitivity in monitoring patients with ovarian carcinoma.

The authors are grateful to Drs J.M. Del Campo, D. Rubio, R. Bodi and G. Encabo (Valle de Hebrón Hospital, Barcelona), Dr H. Cortés-Funes (Doce de Octubre Hospital, Madrid) and Dr S. Zotter (Institute of Pathology, Dresden).

### References

ABE, M. & KUFE, D.W. (1987). Identification of a family of high molecular weight tumor-associated glycoproteins. *J. Immunol.*, **139**, 257.

COLOMER, R., LIANES, P., RUIBAL, A., CORTES-FUNES, H., SALVADOR, L. & HORNEDO, J. (1988). Circulating CA 15-3 levels in patients with breast cancer predict relapse and correlate with metastatic tumor burden. *Proc. Am. Soc. Clin. Oncol.*, **7**, 77.

FRIEDMAN, E., HAYES, D.F. & KUFE, D.W. (1986). Reactivity of monoclonal antibody DF3 with a high molecular weight antigen expressed in human ovarian carcinomas. *Cancer Res.*, **46**, 5189.

GION, M., MIONE, R., DITTADI, R., FASAN, S., PALLINI, A. & BRUSCAGNIN, G. (1986). Evaluation of CA 15/3 serum levels in breast cancer patients. *J. Nucl. Med. Allied Sci.*, **30**, 29.

HAYES, D.F., SEKINE, H., OHNO, T., ABE, M., KEEFE, K. & KUFE, D.W. (1985). Use of a murine monoclonal antibody for detection of circulating plasma DF3 antigen levels in breast cancer patients. *J. Clin. Invest.*, **75**, 1671.

HAYES, D.F., ZURAWSKI, V.R. & KUFE, D.W. (1986). Comparison of circulating CA15-3 and carcinoembryonic antigen levels in patients with breast cancer. *J. Clin. Oncol.*, **4**, 1542.

HILKENS, J., BUIJS, F., HILGERS, J. & 4 others (1984). Monoclonal antibodies against human milk-fat globule membranes detecting differentiation antigens of the mammary gland and its tumors. *Int. J. Cancer*, **34**, 197.

HILKENS, J., KROEZEN, V., BONFRER, J.M.G., DE JONG-BAKKER, M. & BRUNING, P.F. (1986). MAM-6 antigen, a new serum marker for breast cancer monitoring. *Cancer Res.*, **46**, 2582.

JÄGER, W., WILDT, L. & LEYENDECKER, G. (1986). CA 15/3 and CEA serum concentrations in breast cancer patients. In *Clinical Relevance of New Monoclonal Antibodies*, Greten, H. & Klapdor, R. (eds) p. 167. Georg Thieme Verlag: Stuttgart.

KUFE, D., INGHIRAMI, G., ABE, M., HAYES, D., JUSTI-WHEELER, H. & SCHLOM, J. (1984). Differential reactivity of a novel monoclonal antibody (DF3) with human malignant versus benign breast tumors. *Hybridoma*, **3**, 223.

KOLDOVSKY, U. WARGALLA, U., HILKENS, J. & 4 others (1986). CA 125 und MAM 6 in serum und tumor bei ovarialkarzinomen. *Tumor Diagn. Ther.*, **7**, 125.

MOLINA, R., BALLESTA, A.M., FILELLA, X. & 4 others (1986). Estudio de un nuevo marcador tumoral, el CA 15.3 en patologias benigna y neoplasica. *Neoplasia*, **3**, 85.

PAULICK, R., CAFFIER, H. & KAESEMANN, H. (1986). Erste erfahrungen mit dem monoklonalen markersystem CA 15-3 bei mammakarzinompatientinnen. *Tumor Diagn. Ther.*, **7**, 85.

PONS-ANICET, D.M.F., KREBS, B.P., MIRA, R. & NAMER, M. (1987). Value of CA 15:3 in the follow-up of breast cancer patients. *Br. J. Cancer*, **55**, 567.

RUIBAL, A., GENOLLA, J., ROSELL, M. & MORAGAS, M. (1987). El CA 15.3-ELSA serico en patologias no tumorales. *Med. Clin. (Barc.)*, **88**, 476.

SEKINE, H., HAYES, D.F., OHNO, T. & 5 others (1985). Circulating DF3 and CA 125 antigen levels in serum of patients with epithelial ovarian carcinomas. *J. Clin. Oncol.*, **3**, 1355.

STURM, G., SCHMIDT-RHODE, P., SCHULZ, K.-D., FRICK, M. & SOULZ, U. (1987). The diagnostic value of the CA 15-3 determination in the post-care of breast cancer patients: First results of a prospective study. In *New Tumor Markers and their Monoclonal Antibodies*, Klapdor, R. (ed) p. 70. Georg Thieme Verlag: Stuttgart.

YOSHIMOTO, M., AKIYAMA, F., WATANABE, S. & 6 others (1987). Estimates of circulating breast cancer-associated antigen CA 15-3 as a monitoring marker in patients with breast cancer. *Gan To Kagayu Ryoho*, **14**, 2310.

ZOTTER, S., HAGEMAN, P.C., MOOI, J.W., LOSSNITZER, A. & HILGERS, J. (1989). Tissue and tumor distribution of human polymorphic epithelial mucus. *Cancer Res.*, (in the press).